# Fetal body MRI and its application to fetal and neonatal treatment: an illustrative review

**DOI:** 10.1016/S2352-4642(20)30313-8

**Published:** 2021-03-12

**Authors:** Joseph R Davidson, Alena Uus, Jacqueline Matthew, Alexia M Egloff, Maria Deprez, Iain Yardley, Paolo De Coppi, Anna David, Jim Carmichael, Mary A Rutherford

**Affiliations:** Prenatal Cell and Gene Therapy, Elizabeth Garrett Anderson Institute of Women’s Health, University College London, London, UK; UCL Great Ormond Street Institute of Child Health, University College London, London, UK; Stem Cells and Regenerative Medicine; Perinatal Imaging, School of Biomedical Engineering & Imaging Sciences, King’s College London, London, UK; Stem Cells and Regenerative Medicine; Perinatal Imaging, School of Biomedical Engineering & Imaging Sciences, King’s College London, London, UK; Stem Cells and Regenerative Medicine; Perinatal Imaging, School of Biomedical Engineering & Imaging Sciences, King’s College London, London, UK; Stem Cells and Regenerative Medicine; Perinatal Imaging, School of Biomedical Engineering & Imaging Sciences, King’s College London, London, UK; Paediatric Surgery, Evelina London Children’s Hospital, London, UK; UCL Great Ormond Street Institute of Child Health, University College London, London, UK; Specialist Neonatal and Paediatric Surgery, Great Ormond Street Hospital for Children, London, UK; Katholieke Universiteit Leuven, Leuven, Belgium; Prenatal Cell and Gene Therapy, Elizabeth Garrett Anderson Institute of Women’s Health, University College London, London, UK; Fetal Medicine Unit, University College London, London, UK; Paediatric Radiology, Evelina London Children’s Hospital, London, UK; Stem Cells and Regenerative Medicine; Perinatal Imaging, School of Biomedical Engineering & Imaging Sciences, King’s College London, London, UK

## Abstract

This Review depicts the evolving role of MRI in the diagnosis and prognostication of anomalies of the fetal body, here including head and neck, thorax, abdomen and spine. A review of the current literature on the latest developments in antenatal imaging for diagnosis and prognostication of congenital anomalies is coupled with illustrative cases in true radiological planes with viewable three-dimensional video models that show the potential of post-acquisition reconstruction protocols. We discuss the benefits and limitations of fetal MRI, from anomaly detection, to classification and prognostication, and defines the role of imaging in the decision to proceed to fetal intervention, across the breadth of included conditions. We also consider the current capabilities of ultrasound and explore how MRI and ultrasound can complement each other in the future of fetal imaging.

## Introduction

MRI of the fetus is a well-recognised adjunct to ultrasound for the assessment of fetal anomalies. Fetal movements can cause artifacts in MRI and various approaches are used to reduce their negative effect on images, including adjustment of acquisition parameters. However, such adjustment limits the detail derived from the scan (panel). Modern post-acquisition reconstruction techniques allow for motion-correction, three-dimensional (3D) segmentation, and volumetry. The refinement of these techniques has given fetal MRI a well defined role in the assessment of fetal CNS anomalies,^1^ followed by recent success in reconstructing magnetic resonance images to aid in the diagnosis of fetal cardiac lesions.^2^ Advancements have also been made in the assessment of placental pathology using MRI,^3^ with reliable distinction of circulatory systems of the mother and fetus enabling detailed future study of placental development.^4^

Fetal body MRI is now complementary to ultrasound for the assessment of head and neck masses, anterior abdominal wall lesions, and thoracic or abdominal pathology. Many of these body regions have been particularly challenging to image, owing to fetal movement or the deformable nature of the cavity wall. This problem has limited the role of MRI to the assessment of contiguous but misaligned two-dimensional (2D) slices, which has clear disadvantages in the assessment of subtle abnormalities and undersells the potential of MRI for providing 3D data. This illustrative Review summarises current advances in MRI for malformations of the fetal body that might be amenable to fetal or early postnatal intervention. The widely recognised gold standard of fetal ultrasound is used as a comparator, and the circumstances are shown in which the two methods can complement each other in the assessment of challenging cases.

This Review covers lesions of the head and neck, thorax, abdomen and pelvis, and spine, for which MRI has a less established role than for lesions of the fetal brain or heart. Each section contains several illustrative case reports with relevant antenatal history and information derived from images. New image reconstruction techniques are shown that could further increase the benefits of MRI; specifically illustrative examples of deformable slice-to-volume registration, which allows reconstruction of high-resolution 3D images in modifiable planes and 3D segmentation to improve accuracy of volume measurement (appendix p 1).^5^

## Head and neck masses

**Figure 1:**
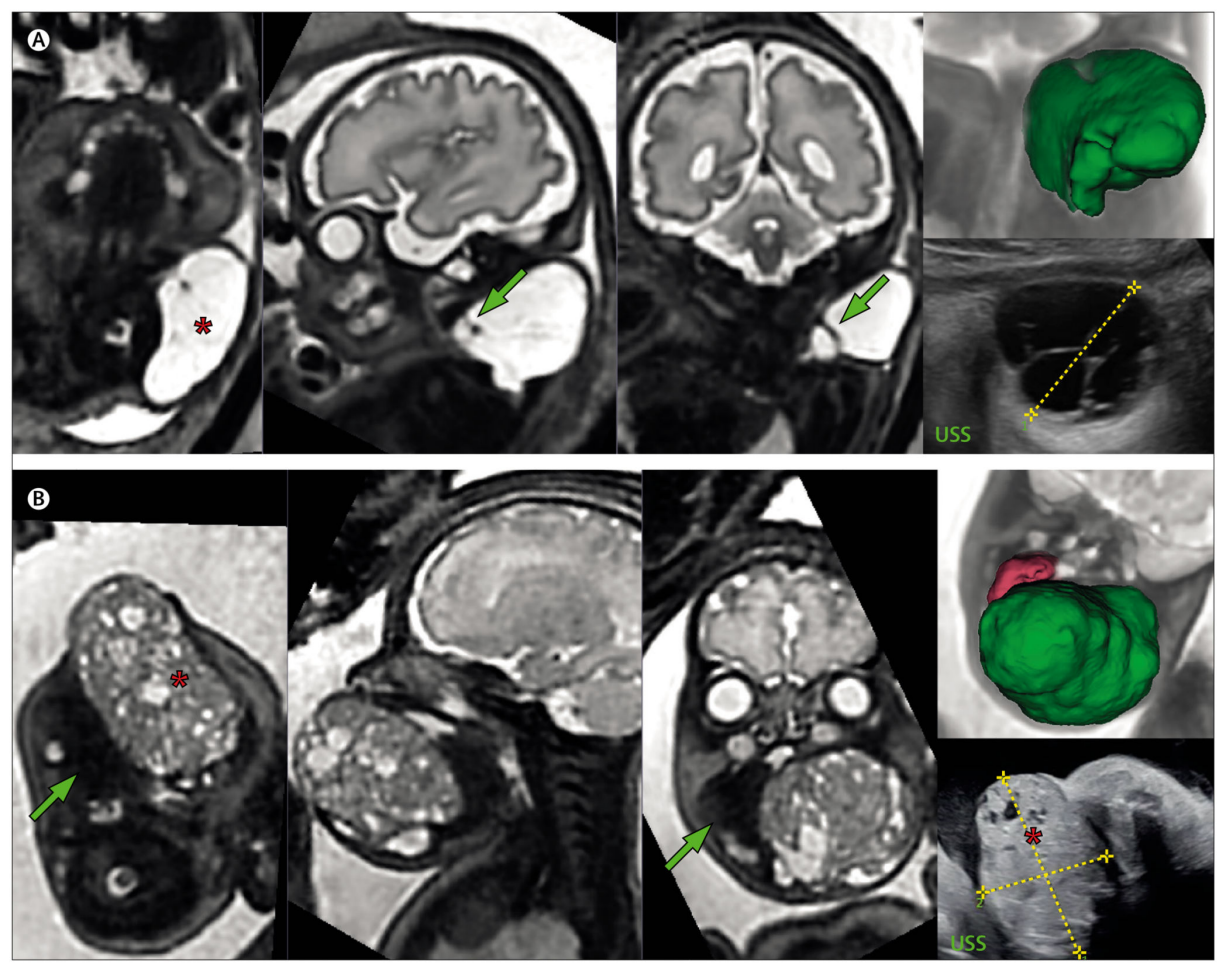
T2-weighted MRIs of two fetuses with neck masses (A) Fetus at 31 weeks and 6 days’ gestation with a lymphatic malformation (marked by an asterisk) of the posterolateral left neck, with arrows pointing to internal septations. The 3D model (top right) shows the lesion (green) lying separate to the fetal airway, major vessels, and CNS structures. The corresponding fetal ultrasound is shown bottom right. (B) Fetus at 33 weeks and 4 days’ gestation with an oropharyngeal teratoma (marked by an asterisk) in relation to the tongue (green arrow). The sagittal and coronal views in the two central images show the relationship to the fetal airway. The corresponding fetal ultrasound is shown bottom right. The 3D model (top right) shows the 85 cm^3^ tumour mass (green) and tongue (pink). 3D=three-dimensional. USS=ultrasound scan.

Most antenatally diagnosed neck masses are either lymphatic malformations (figure 1A) or teratomas (figure 1B), with a prevalence of one per 10 000–30 000 pregnancies.^6^ The predominant concern is that of neonatal airway compromise, and the advent of ex-utero intrapartum treatment has enabled life-saving measures for the fetus with a critical airway. These measures involve doing a carefully planned airway intervention while maintaining feto–placental circulation until the fetus can be separated from the umbilical cord to complete the caesarean section delivery. Identifying which fetuses might need ex-utero intrapartum treatment remains a challenge, and requires referral to specialised centres that can coordinate the necessary investigation and intervention.^7^ Evaluation by fetoscopy is an alternative means of imaging to assess the fetal airway; however, the associated procedural risks should be recognised.^8^ However, although ex-utero intrapartum treatment in specialist centres has proved to be successful, it is an invasive procedure with substantial risks to both mother and fetus. Maternal and fetal factors, and amount of institutional expertise, should be considered, with the availability of accurate imaging clearly being important.

For fetal neck masses, MRI provides a clear advantage over conventional ultrasound for assessing tumour extension and giving a 3D visualisation of the tumour’s relation to the airway (figure 1 and video 1). MRI can distinguish between lymphatic malformations and teratomas by using T1 and T2 contrast weightings that identify fluid, haemorrhage, and fat, enabling anatomical relations to be shown precisely. T1-weighted MRI is also able to reliably identify thyroid tissue,^9^ and this is important if considering the need for a tracheostomy, given that passage of the tumour anterior to the trachea might render this procedure infeasible. A large retrospective series showed that fetuses requiring ex-utero intrapartum treatment could be predicted using MRI-measured dimensions of polyhydramnios and evidence of mass effect on the trachea, with a sensitivity of 95% and a specificity of 80%, although tumour size itself was not a predictive factor.^10^

For assessing bony defects such as orofacial clefts, the value of MRI is generally surpassed by that of 3D ultrasound, because the latter gives a detailed depiction of the fetal face. However, MRI is still of use in suspected cases,^11^ because it can be more sensitive than 3D ultrasound in detecting cases of isolated cleft palate,^12^ for which the absence of an associated labial defect could render diagnosis by ultrasound difficult.

### Illustrative cases

Figure 1A shows the T2-weighted MRI of a fetus of 31 weeks and 6 days’ gestation with a lymphatic malformation of the posterolateral neck. The fetal MRI was able to exclude the diagnosis of teratoma by ruling out any solid components of the lesion, show the multi-compartmental involvement (including the carotid space), and importantly, show the preservation of the fetal airway. The baby was born asymptomatic at term and remains under observational follow-up at 2 years of age.

Figure 1B shows the T2-weighted MRI of a fetus at 33 weeks and four days’ gestation with a pharyngeal teratoma. 3D reconstruction measured tumour volume at 85 cm^3^. Lateral displacement of the tongue and effacement of the oropharyngeal cavity were appreciable on the fetal ultrasound scan. MRI was able to establish the margins of the fetal airway and supported the decision to give ex-utero intrapartum treatment, in which video-assisted oral intubation was done before neonatal tracheostomy. Detailed illustration of the 3D reconstruction of these two lesions is provided in videos 1 and 2.

## Thoracic lesions

### Lesions of the lung

**Figure 2:**
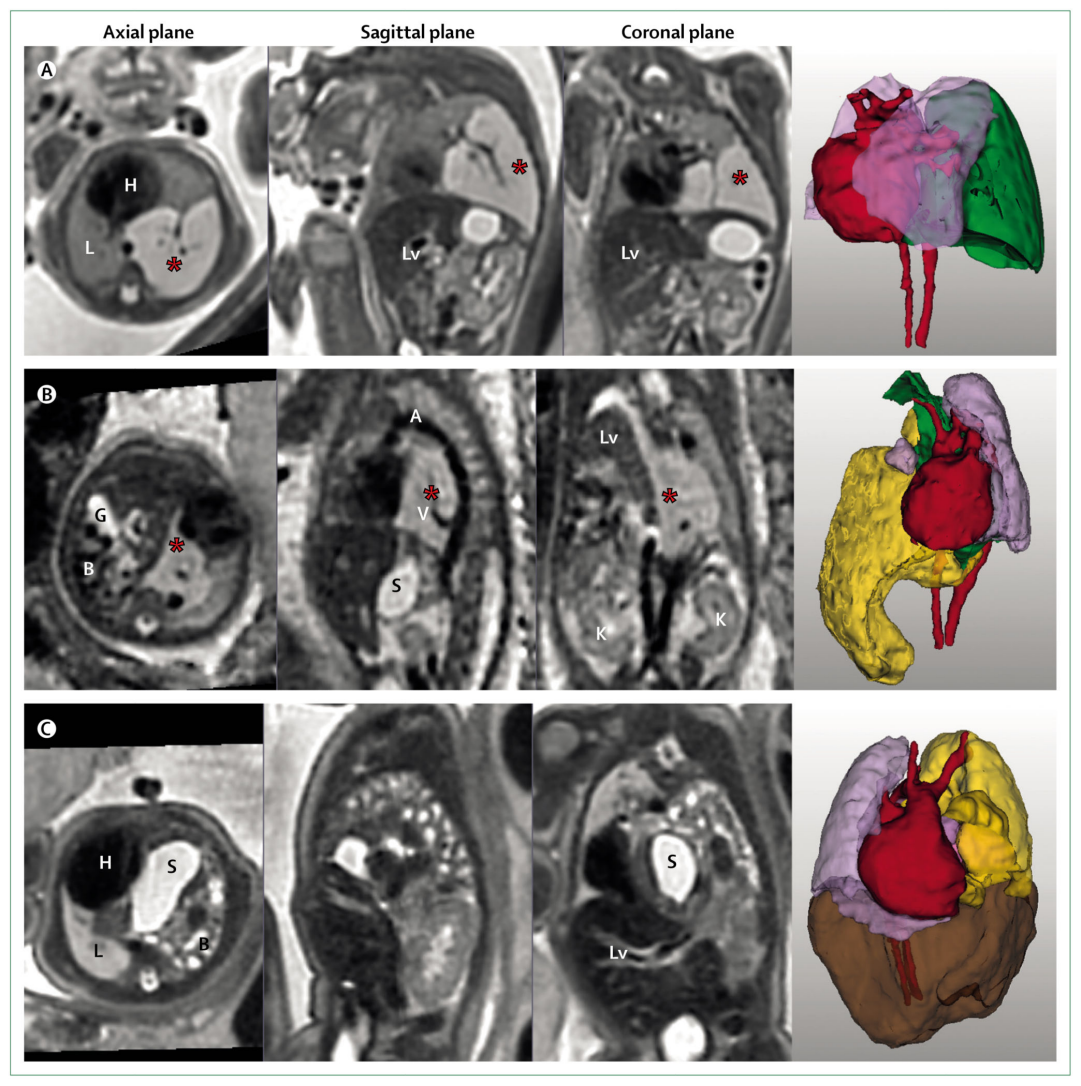
T2-weighted MRI and three-dimensional models of three fetuses with lesions of the thorax (A) Fetus of 24 weeks’ gestation with a lesion of left lower lung showing a microcystic congenital pulmonary airway malformation of the left lower lobe (marked by an asterisk), also shown in 3D (green), with heart (red) and lungs (lilac). (B) Fetus of 24 weeks’ gestation with bronchopulmonary sequestration (marked by an asterisk) and right congenital diaphragmatic hernia. Reorientation to true sagittal shows a feeding vessel arising from the aorta. Note the right-sided diaphragmatic hernia containing the bowel (yellow), gallbladder, and stomach. Kidneys are shown in the coronal plane. 3D models show bronchopulmonary sequestration (green), lungs (lilac), and heart (red). (C) Fetus of 33 weeks’ gestation with a left-sided congenital diaphragmatic hernia, containing stomach and intestine (yellow), and liver (brown) within the hernia, and showing the heart (red) and lung (lilac) in relation to hernial content. 3D=three-dimensional. A=aorta. B=bowel. G=gallbladder. H=heart. K=kidney. L=lung. Lv=Liver. S=stomach. V=vessel.

Bronchopulmonary foregut malformations include intestinal duplications, bronchogenic cysts, bronchial atresia, congenital pulmonary airway malformations (figure 2A), and bronchopulmonary sequestrations (figure 2B). Prevalence has previously been estimated at 1 per 20 000; however, improved detection using high-resolution ultrasound suggests a true prevalence closer to one per 3000.^13^ Congenital pulmonary airway malformations can be solid or cystic; fetal ultrasound can reasonably reliably define these lesions and differentiate them from broncho-pulmonary sequestrations on the basis of a characteristic systemic arterial blood supply identified using colour doppler (figure 2B). The possibility of a hybrid lesion, with cystic architecture and a systemic feeding vessel, is also well recognised.

Generally, lung lesions are asymptomatic during fetal development; however, large lesions can lead to hydrops caused by compression of adjacent structures, with an extremely poor prognosis if left untreated.^14,15^ Postnatally, many lesions are often asymptomatic, and can even involute, but some will develop infection, which is an indication for resection.^16^ Observation of the asymptomatic lesion is a contentious issue, and some surgeons believe that resection in lesions affected by infection is particularly challenging to do and therefore carries an increased risk of surgical complications.^17^ The amount and quality of remaining normal lung is a key question to ask of imaging, to help direct antenatal counselling, and here MRI-derived volumetric information could help with prognosis and surgical planning (videos 3–5). Furthermore, MRI could be better than ultrasound for distinguishing between normal and abnormal lung tissue, and in making other diagnoses such as diaphragmatic hernia—particularly in late gestation, when doing so with ultrasound is challenging. Retrospective studies from large-volume referral centres showed that a defined diagnosis was reclassified after MRI in up to 50% of cases,^18^ suggesting that MRI has improved diagnostic accuracy compared with ultrasound and can help with planning an appropriate delivery location if neonatal respiratory compromise is suspected.

### Congenital diaphragmatic hernia

Congenital diaphragmatic hernia has an estimated prevalence of one per 2000–4000 pregnancies and results in a herniation of abdominal viscera into the chest. Consequently, the developing lungs are abnormal in terms of airspace volume and vasculature, on both the affected and the contralateral side. Postnatal morbidity and mortality result from ventilation difficulties, pulmonary hypertension, and cardiac failure.

In expert centres, antenatal measures of disease severity have been shown to be as accurate as measures taken after birth,^19^ affirming the importance of the standardisation of fetal assessment. Ultrasound can predict outcomes in congenital diaphragmatic hernia with reasonable accuracy using the ultrasound measurement of observed-to-expected lung-to-head ratio. This ratio takes the size of the contralateral lung in a single plane as a surrogate measurement for total lung volume, and remains the most commonly used antenatal prognostic indicator.^20^ Several previous studies have failed to show the superiority of MRI in predicting outcome compared with observed-to-expected lung-to-head ratio;^20,21^ however, MRI is able to give a more objective and reproducible estimate of the total lung volume (figure 2),^22,23^ which has been shown to be a defining measure of long-term postnatal oxygen requirement.^24^ Although fetal interventions such as fetoscopic endotracheal occlusion are done on the basis of ultrasound measurement of observed-to-expected lung-to-head ratio, MRI might soon become a more accurate way to stratify patients with congenital diaphragmatic hernia.^25^

**Figure 3:**
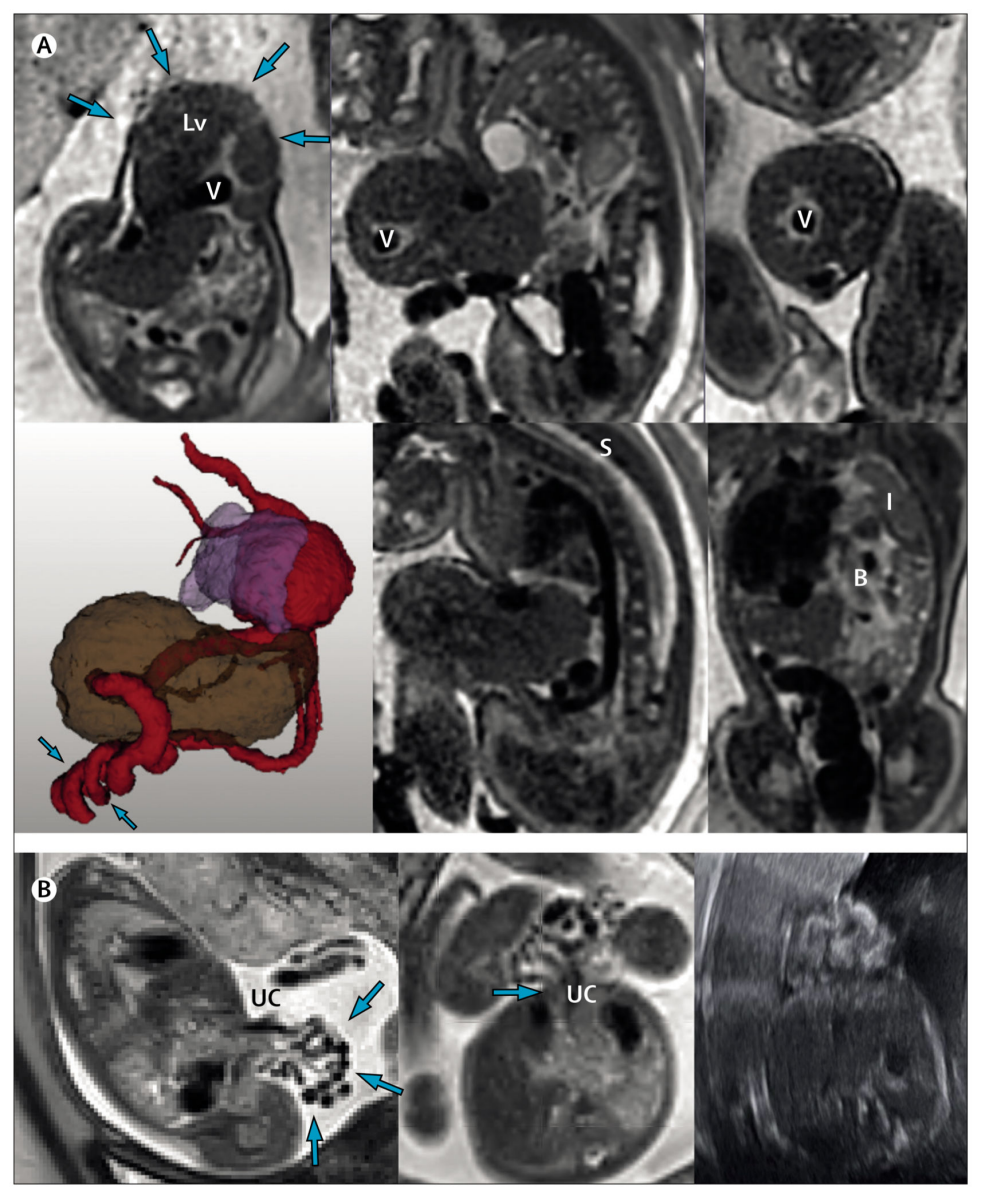
MRIs of two fetuses with abdominal wall defects (A) Fetus at 34 weeks and 6 days of gestation with exomphalos (arrows on axial image, top left). The 3D image shows that the exomphalos contains the liver (brown) and the prominent umbilical vein (red) follows a twisting and irregular course (arrows; lung shown in lilac). The lower middle panel shows marked angulation of the thoracic spine. Bowel in the associated diaphragmatic hernia is shown in the lower right image with the compressed left lung. (B) Gastroschisis, with no sign of bowel dilatation on the MRI. The exteriorised bowel (arrows, left image) and insertion (arrow, central image) of umbilical cord are shown. Image on the right shows the referring ultrasound scan. B=bowel. I=lung. Lv=liver. S=spine. UC=umbilical cord. V=vein.

Most 3D-image reconstruction techniques for assessing the fetal lung have been able to detect only severe cases of pulmonary hypoplasia.^26^ However, novel motion-correction and reconstruction approaches (eg, deformable slice-to-volume registration reconstruction) can reliably produce volumetric measurement, even from motion corrupted sequences (appendix p 1, figure 2, figure 3A, videos 6–7).^27^ Work to produce normative lung volumes throughout gestation is ongoing; as such, the prognostic relevance of these measurements is currently anecdotal. Spatial anatomical relations are shown much more clearly on fetal MRI than ultrasound, thereby allowing precise location of the lung, liver, stomach, and bowel (figure 2). This capacity has important prognostic implications, because the presence of liver within a hernia is well recognised as a poor prognostic marker.^23^ The dimensions of the hernia defect are important determinants of operative technique and postoperative course, and MRI has even been used to produce 3D-bioprinted patches for defect coverage.^28^ Looking to the future, accurate sizing of a patch would be an important step in the preparation of tissue-engineered solutions.^29^

### Illustrative cases

Figure 2A shows the T2-weighted MRI of a 24 weeks’ gestation fetus with a lesion of the left lower lung. Antenatal diagnosis was microcystic congenital pulmonary airway malformation. The lesion volume was measured (17·26 cm^3^) along with the remaining total lung volume (12·82 cm^3^). There was displacement of the mediastinum, including the heart, into the right hemithorax; however, notably there was no hydrops. Detailed illustrations of the 3D reconstructed image and segmentations are in videos 3–4. This lesion seemed to resolve on serial fetal ultrasound and postnatal chest x-ray, the infant remains asymptomatic during ongoing follow-up.

Figure 2B shows a fetus at 25 weeks and 2 days’ gestation with a T2 hyperintense lesion behind the heart (bright signal on T2-weighted images consistent with a high water content). Reorientation of planes using deformable slice-to-volume registration reconstruction clearly showed a systemic feeding vessel arising from the descending aorta, confirming the diagnosis of bronchopulmonary sequestrations. A right-sided diaphragmatic hernia was also noted, with liver and bowel herniating into the right chest. This combination of pathology results in a low total lung volume (7·82 cm^3^). Detailed illustrations of the 3D reconstructed image and segmentations can be seen in video 5. The baby was born at 30 weeks’ gestation but unfortunately died on the first day of life.

Figure 2C shows a 33 weeks’ gestation fetus with left-sided congenital diaphragmatic hernia containing stomach, bowel, spleen, and liver, and with partial herniation of the kidney. A markedly hypoplastic lung can be seen on the left side. The total lung volume calculated from segmentation is 23·47 cm^3^. The patient had postnatal repair on day 6 of life and is currently being followed-up at age 3 years. Detailed illustrations of the 3D reconstructed image and segmentations can be seen in videos 6–7.

### Oesophageal atresia

**Figure 4:**
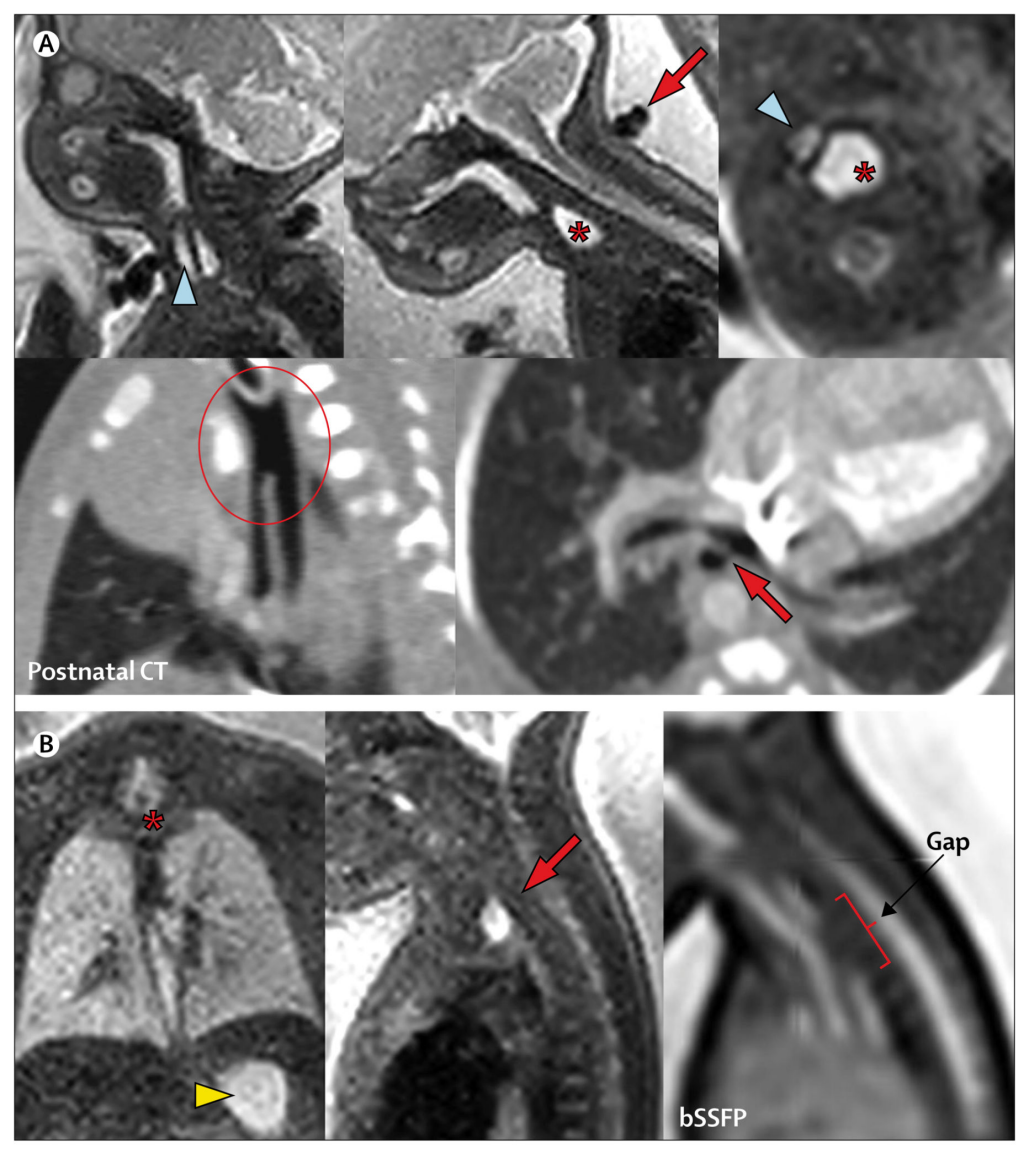
MRIs of two fetuses with oesophageal atresia (A) Fetus at 32 weeks and 5 days of gestation has a dilated upper pouch (marked by an asterisk), displacing the trachea (blue arrowhead) in the axial view (upper right image). A two-vessel umbilical cord (red arrow) can be seen in the sagittal plane (top middle image). The postnatal CT (lower two images) shows a long-segment common tracheoesophageal channel (encircled, left) and distal fistula arising from the left main bronchus (red arrow, right). (B) Fetus at 30 weeks and 5 days of gestation has a dilated upper pouch (marked by an asterisk in the left image and an arrow in the central image) and normal stomach (arrowhead), suggesting a distal fistula. Gap measurement is possible in the sagittal image (right). bSSFP=balanced steady-state free precession MRI.

Accurate antenatal diagnosis of oesophageal atresia is of particular interest to surgeons advocating centralisation of care for this condition. Oesophageal atresia (with or without tracheoesophageal fistula) is a spectrum of disorders that has an overall prevalence of about one in 3000. There is usually an associated fistula, ligation of which is necessary in the first days of life, ideally with simultaneous repair of the atresia. Currently, fetal ultrasound manages to identify oesophageal atresia in less than a third of cases. Findings of a small stomach and polyhydramnios elevate the index of suspicion, but confirmation of a oesophageal atresia diagnosis is rarely possible because of the low specificity of these findings, which can be related to oropharyngeal or mediastinal masses, poor fetal swallowing, or simply a normal variant.^30^ A systematic review found that MRI used in conjunction with ultrasound provided sensitivity greater than 94% and specificity of 89%.^31^ Findings of dilated upper oesophagus with bowing of the trachea can be readily seen on MRI (figure 4A) and have a sensitivity of 91% and specificity of 100% in cases suspected on fetal ultrasound.^32^

When the authors of this Review used post-acquisition reconstruction, we were unable to improve upon the diagnostic accuracy of 2D MRI images because the averaging process of reconstruction algorithms can potentially reduce any appearance of distension in an organ with peristalsis. The Review authors found that with improved image acquisition (figure 4B), the distal oesophageal lumen (recognised to be indicative of a tracheoesophageal fistula) can be seen. This could enable prediction of a long-gap oesophageal atresia, which often requires oesophageal replacement using a conduit, a procedure with both short-term and long-term morbidity.^33,34^ The Review authors anticipate that reliable and accurate diagnosis of such cases could create a role for tissue-engineered constructs, similar to what has been achieved with congenital diaphragmatic hernias.^29^

### Illustrative cases

Figure 4 shows fetal MRI of two fetuses with oesophageal atresia. Figure 4A shows a fetus at 32 weeks and 5 days’ gestation with complex foregut anatomy; the fetal MRI shows a dilated oesophageal pouch displacing and bowing the trachea. The postnatal CT imaging shown alongside shows the complex common channel of trachea and oesophagus, and the distal oesophageal fistula relating to the left main bronchus, which is hypoplastic. The complexity of the lesion prompted a redirection of care and the infant died on the second day of life. Figure 4B shows a conventional type C atresia (oesophageal atresia with distal fistula) in a fetus at 32 weeks and 5 days’ gestation. The dilated upper pouch is best seen on solitary coronal slices of the MRI (without reconstruction), and the balanced steady-state free precession sequence sagittal view enables visualisation of the gap between the two ends of the oesophagus (three vertebral bodies). The infant had neonatal repair via thoracotomy and was well at last follow-up at age 3 years.

## Lesions of the abdomen and pelvis

### Abdominopelvic cyst

Abdominopelvic cysts are a common finding on fetal ultrasound scans, documented in approximately one per 1000 pregnancies, and are typically ovarian in origin. The clinical postnatal outcomes of antenatally diagnosed cysts have been well described; many will involute during antenatal or early postnatal life, and they are generally managed expectantly.^35^ However, a small proportion enlarge or cause problems owing to mass effect,^36^ and non-ovarian cysts are still challenging to diagnose on ultrasound scans.^37^ These rarer diagnoses might require early specialist referral—eg, in cases of choledochal malformation, lymphatic malformation, or fetal tumour. Antenatal counselling requires reliable delineation of the cyst and its spatial and anatomical relations. A small series suggested that the spatial resolution of MRI in 3D allows for more accurate anatomical delineation and improved diagnostic accuracy compared with ultrasound.^38^ However, caution should be used regarding absolute reliance on MRI, because two cases in the series were incorrectly reclassified after MRI, including a case of adrenal haemorrhage. It is worth noting that modern T2-weighted MRI sequences are haemorrhage-sensitive, but although widely used in placental MRI research,^39^ these sequences have not been optimised for fetal clinical application.

### Abdominal wall defects

Fetal ultrasound is usually able to diagnose, and differentiate between, common abdominal wall defects, exomphalos, and gastroschisis. This distinction is important, because the antenatal counselling and post-natal outcomes of each of these conditions is markedly different. Exomphalos, with a prevalence of one per 13 000, is commonly associated with other midline structural defects that can be shown on ultrasound (eg, cardiac, renal, spinal, diaphragmatic; note the associated congenital diaphragmatic hernia shown in figure 3A). The clustering of several lesions can lead to a missed diagnosis if only ultrasound is used. In a retrospective case control study of individuals with complex exomphalos, MRI had an improved detection rate compared with fetal ultrasound and detected additional anomalies postnatally in twice as many fetuses.^40^ Accurate sizing of the defect is another advantage of MRI, because defects larger than 5 cm can be associated with prolonged respiratory morbidity, and to morbidity related to feeding associated with the process of restoring abdominal organs and achieving abdominal closure.

Gastroschisis, by contrast, is usually an isolated lesion and occurs in one in every 6000–10 000 pregnancies, with substantial regional variation. Although outcomes are generally good, up to 25% of affected neonates can develop substantial morbidity, although predicting this antenatally is notoriously challenging.^41^ Complex gastroschisis can involve bowel necrosis, atresia, and dysmotility, thought to be due to a combination of amniotic fluid exposure and defect constriction.^42^ Sudden onset of dilatation of the stomach, or intestine, or both, or polyhydramnios, are used as surrogate markers of bowel atresia on fetal ultrasound, and data from a prospective prognostic study support the value of serial 2D measurements in the prediction of complex cases.^43^ The ability of MRI to differentiate bowel content (ie, meconium showing bright on T1-weighted MRI, or dark on T2-weighted MRI, figure 3B) might predict an associated atresia and enable an estimate of bowel viability.^44^

Pulmonary hypoplasia can occur in both gastroschisis and exomphalos, and tends to be associated with defect size in isolated defects, but is also related to the associated anomalies in exomphalos.^45^ Long-term morbidity correlated to lung volumes and fetal lung volume assessment by MRI might be useful to inform antenatal counselling.

### Illustrative cases

Figure 3A shows a fetus at 34 weeks and 6 days of gestation with exomphalos. The abdominal wall defect, measured in multiple planes, is approximately 5 cm in width and there is substantial herniation of the liver and a prominent and irregular-coursing umbilical vein (highlighted on the 3D model). Associated spinal angulation and a severe left congenital diaphragmatic hernia were also present, with intrathoracic loops of bowel, spleen, and stomach. The total lung volume was calculated as 16·61 cm^3^. The parents chose palliative care after delivery. Detailed illustrations of the 3D reconstructed image and segmentations can be seen in videos 8–9.

Figure 3B shows a fetus at 24 weeks’ gestation with gastroschisis. Loops of bowel outside of the abdominal cavity and without membrane coverage are clearly shown, and the defect can be seen to originate to the right of the insertion of the umbilical cord. This pregnancy was induced (per protocol) at 37 weeks and 5 days’ gestation, the baby had primary closure at the cotside on the first day of life, and was well at follow-up more than 2 years later.

### Bowel atresia, meconium peritonitis, and anorectal malformation

Intestinal atresia is found in approximately one per 3000 pregnancies and is associated with an underlying syndrome in an estimated 20% of cases.^46^ Fetal ultrasound findings of echogenic and dilated bowel are associated with bowel atresia, but these findings have such low specificity that their predictive value is not clinically useful.^47^ MRI can show progression of meconium through the fetal bowel across gestation.^48^ Features of non-progression of meconium therefore suggest a congenital obstruction, and signs of increased bowel volume can help to identify the extent of atresia.^49^ A further, albeit rare, presentation of intestinal atresia is antenatal perforation leading to meconium peritonitis. Meconium peritonitis is occasionally seen in fetuses with cystic fibrosis, and such patients might not require any postnatal surgical intervention. However, high mortality is associated with cases of intestinal volvulus in which mesenteric ischaemia is untreatable antenatally. Although MRI is less able than ultrasound to detect classical peritoneal calcifications,^50^ a partly-blinded comparative study suggested that MRI detection of proximal dilatation with micro-colorectum is a more reliable indicator than ultrasound of fetuses requiring postnatal surgical intervention.^51^

Anorectal malformations have a prevalence of approximately one per 5000 pregnancies and represent a wide spectrum of anomalies, ranging from anterior anus and perineal fistula through to persistent cloaca. Many of these malformations are difficult to diagnose using any form of fetal imaging. This difficulty might result from an inadequate magnetic resonance signal and ultrasound acoustic shadowing of the fetal pelvis, plus (in many cases) an absence of antenatal diagnostic features, such as rectal dilatation. However, the spatial relationship of urogenital and intestinal structures in the fetal pelvis have enabled cloacal anomalies to be diagnosed with reasonable reliability using MRI.^52^ Imaging findings might also include abnormal signal within the bowel, secondary to a mixing of urine and meconium, alongside any associated Müllerian abnormalities.

### Illustrative case

The appendix (p 2), shows a fetus MRI-scanned primarily for cardiac concerns (ie, suspected ventricular septal defect and coarctation of the aorta identified on fetal echocardiography). Findings on review of the MRI of the abdomen revealed dilated distal bowel loops, suggesting a distal obstruction; which was confirmed postnatally as an anorectal malformation (with a fistula to the bulbar urethra). Retrospective review of the images showed possible anterior deflection of the rectum; however, the subtlety of this finding serves as evidence of how challenging antenatal diagnosis of anorectal malformation can be. At initial clinical reporting, note was made that the meconium signal (ie, fetal colon) was seen only on the left side of the abdomen and the duodenum did not take a retroperitoneal course in the third part (appendix p 2), together suggesting intestinal malrotation, which was confirmed at laparotomy on the first day of life. Associated with the malrotation, a preduodenal portal vein can also be seen (appendix p 2).

## Spinal lesions

### Sacrococcygeal teratoma

With a prevalence of around one per 40 000 livebirths, sacrococcygeal teratoma is the most common type of tumour in neonates. As a teratoma, such lesions often contain calcified elements that cause acoustic shadowing, which can obscure sonographic views required to delineate the internal portion of the tumour. This problem has clinical relevance, because predominantly intrapelvic lesions have a poor prognosis.^53^ Defining the consistency of the overall tumour regarding its solid or cystic proportion also carries important prognostic relevance in terms of tumour grade; solid, hypervascularised masses can lead to fetal cardiac failure, and are candidates for fetal intervention involving debulking or expedited delivery.^54–56^ MRI is useful for showing the internal characteristics of the tumour, its intrapelvic extent, and involvement of the spinal canal.^57^ Also, MRI is particularly useful for distinguishing the fat tissue of the mass from the surrounding pelvic side wall and the meconium content of the fetal bowel (appendix p 3 and video 10). Because tumours often enlarge rapidly during fetal development, clearly there is an ongoing role for interval imaging in monitoring evolving compression or rapid growth, and in the most severe cases fetal intervention or elective preterm delivery could be required.^57,58^

### Illustrative case

The appendix p 3 and video 10, shows a fetus at 24 weeks and 5 days of gestation with a sacrococcygeal teratoma. The predominantly cystic structure can be seen extending into the pelvis with little external aspect (type IV lesion); the 3D model shows the close proximity to the rectum, which is important in surgical planning. Such lesions would be extremely difficult to categorise on ultrasound because of acoustic shadowing.

### Spina bifida

Open spina bifida has an estimated prevalence of one per 2000 pregnancies, with meningomyelocele being the most common variant. Fetal ultrasound diagnosis is highly accurate. The associated hindbrain herniation (ie, Chiari malformation) leads to a typical banana-shaped cerebellum with a lemon-shaped sculpting of the skull, both commonly seen on mid-gestation ultrasound. The vertebral defect and herniated content are usually also seen, and sonographic assessment of lower limb and bladder function can also be made. First trimester ultrasound diagnosis is also feasible using the so-called crash sign, which describes posterior displacement and deformation of the mesencephalon against the occipital bone in axial view.^59^

**Figure 5:**
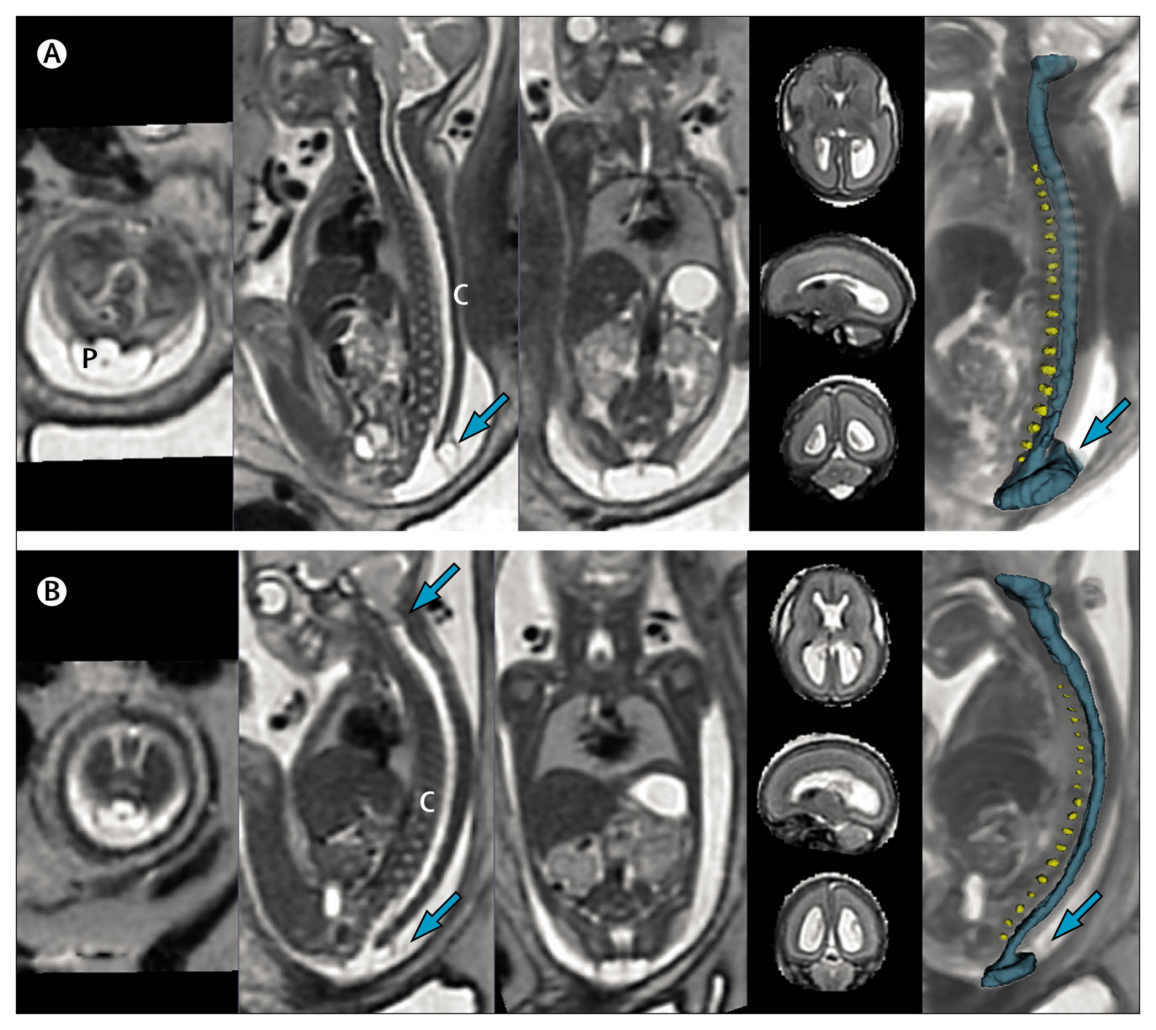
MRIs of two fetuses with spina bifida (A) Open spina bifida (non-eligible for fetal surgery). First and second sacral vertebral defect with cord, dorsal sac (arrow), and placode. The three-dimensional model shows intervertebral discs (yellow) and spinal canal (blue). (B) Open spina bifida (eligible for fetal surgery). Low sacral defect with hindbrain herniation (upper arrow on sagittal view). C=spinal cord. P=placode.

In an era of fetal surgery, defining defect extent and other associated abnormalities is important in the selection of fetuses for whom fetal meningomyelocele closure will bring functional benefit.^60^ Here fetal MRI allows the spinal defect extent to be assessed with similar accuracy to ultrasound (figure 5 and videos 11–12).^61^ However, compared with ultrasound, MRI has the additional benefit of better defining any associated intracranial developmental anomalies that can confer a poor prognosis, such as agenesis of the corpus callosum or cortical migration anomalies. Postoperative MRI to assess resolution of Chiari malformation is now used as a quality measure for surgery and enables monitoring of fetal brain development, improvement in cerebrospinal fluid volume in the posterior fossa, resolution of hindbrain herniation, and improvement of ventricular dilatation.^62^

MRI is also useful for diagnosing, and to counsel the parents of, a fetus with other spinal lesions such as hemivertebra or VACTERL association (which typically involves at least three of the following: vertebral defects, anal atresia, cardiac defects, tracheo-esophageal fistula, renal anomalies, and limb abnormalities). MRI can be used to predict long-term lower limb, bladder, and bowel function. MRI is also a useful adjunct to a fetal ultrasound that has detected closed neural tube defects, in providing further characterisation of spinal cord anomalies, such as tethering.^63^

### Illustrative cases

Figure 5 shows two fetuses with meningomyelocele who were assessed for fetal surgery. Figure 5A is a deformable slice-to-volume registration reconstruction MRI done at 26 weeks’ gestation, the sacral defect is shown at the first and second sacral vertebra with sac and placode extending beyond the skin level. The brain is also seen in the same scan, the absence of hindbrain herniation and reasonable normal appearances of the hindbrain and posterior fossa meant that this fetus did not meet the eligibility criteria for fetal surgery and was treated postnatally. Detailed illustration and segmentations are in videos 11 and 12. By contrast, figure 5B shows MRI images of a fetus at 23 weeks and 1 days of gestation, in which hindbrain herniation can be seen. Fetal surgery was done at 24 weeks’ gestation, with subsequent resolution of the hindbrain herniation before delivery, after follow-up fetal MRI.

## Lesions of the kidneys and urinary tract

Hydronephrosis is a common finding on fetal imaging, with a prevalence of 1% of all pregnancies. The condition might be self-limiting and idiopathic; however, it can also be a consequence of several different abnormalities of the renal collecting system and ureter, or of lower urinary tract obstruction. Posterior urethral valves can be shown on fetal MRI, with the characteristic appearance of a dilated posterior urethra. A finding of a dilated bladder, or bilateral antenatal hydronephrosis, or both, requires careful investigation, because fetal megacystis can present in several chromosomal abnormalities.^64^ Lower urinary tract obstruction can result in a reduced amniotic fluid volume, which can make ultrasound examination of the underlying lesion difficult. However, the paucity of amniotic fluid is of little consequence in MRI acquisition and reconstruction. Management is complex, because a distended bladder in the first trimester often either empties spontaneously or resolves after a single drainage guided by ultrasound^65^ and the benefit of more invasive treatments (eg, bladder shunting in lower urinary tract obstruction) is unclear, as shown by the PLUTO trial.^66^ There is recognition among fetal interventionalists that better patient selection for bladder shunting is needed. This selection requires antenatal assessment of both renal parenchyma and amniotic fluid volume, which can be done with modern MRI acquisition and processing.^67,68^ Although fetal MRI is not a gold standard for diagnosis, it is a recognised useful adjunct to fetal ultrasound in the evaluation of identified pathology.^69,70^

Fetal MRI has further prognostic value, in assessing the renal parenchyma in detail for the presence of cysts and parenchymal dysplasia, detailed in the illustrative cases that follow. Furthermore, MRI can help to measure lung volumes in the context of severe oligohydramnios, in which lung hypoplasia presents an extreme risk to a neonate’s survival.^71^ Diagnosis of bilateral renal agenesis is a further condition in which MRI might be useful, because on ultrasound, in the presence of anhydramnios, the adrenal glands can be mistaken for renal tissue, whereas MRI is able to better indicate that the kidneys are absent.^72^

### Illustrative cases

Two examples of hydronephrosis and one of unilateral multicystic dysplastic kidney are used. The appendix p 4 shows a 30 weeks’ gestation fetus with unilateral hydronephrosis. Some loss of the corticomedullary differentiation can be seen in the affected left kidney when compared with the right kidney. This pregnancy was referred with a known cardiac diagnosis that continued to be managed at the referring centre. In a fetus at 33 weeks and 6 days’ gestation (appendix p 4), the right kidney shows calyceal clubbing and thinning of the renal parenchyma (both hallmarks of severe hydronephrosis) and the left kidney shows features of moderate disease similar to the first fetus. Detailed 3D reconstruction can be seen in video 13. This pregnancy was completed at a referring centre so the postnatal outcome is inaccessible for this infant. The appendix (p 4) shows a left-sided unilateral multicystic dysplastic kidney in a fetus at 20 weeks and 6 days’ gestation, and the accompanying image is of the referring ultrasound scan, which was unable to exclude a dilated left collecting system because of acoustic shadowing. The kidney is made up entirely of communicating cysts, and on MRI clearly differs from the hydronephrotic kidneys seen in the previous images. The 3D reconstruction is shown in video 14. The patient remains under follow-up with noted compensatory hypertrophy of the right kidney and awaits functional nuclear imaging.

## Conclusions

Fetal MRI has been shown to complement fetal ultrasound in many conditions, and to be superior to ultrasound in others because of its precise delineation of body anatomy. Wider adoption of fetal MRI could accelerate its validated use for the diagnosis and investigation of many fetal anomalies, such as those discussed in this Review, and facilitate routine referrals through established pathways. MRI also overcomes several technical limitations of ultrasound, such as atypical fetal position, reduced amniotic fluid volume, or high maternal body-mass index.

Our unit has experience in doing postacquisition reconstruction on externally acquired MRI images; however, these outputs depend on the quality of the original images and acquisition of sufficient image stacks in all three planes. Although the deformable slice-to-volume registration reconstruction tool is already available for download, further technical developments are needed to support its full clinical translation, including robustness to fetal motion, and evaluation of the effects on volumetric measurements. It is important to reiterate that reconstructed volumes should be used in conjunction with 2D images for diagnosis, because the averaging process might occlude visualisation of dynamic physiological processes such as peristalsis or cause blurring owing to peristaltic motion.

A benefit common to all conditions discussed in this Review is the communicability of the images gained with MRI; 3D ultrasound gives excellent spatial resolution of craniofacial and skeletal anomalies but (for non-expert sonographers) involves greater difficulty in interpreting soft tissue lesions within the thorax and abdomen. The cross-sectional images obtained on conventional MRI are readily interpretable for a surgeon, and with progress made in 3D reconstruction, are likely to guide treatment with increased precision.

The superiority of MRI to ultrasound in prognostication is supported by data in only a few conditions at present, reflecting the current early stage of development of fetal body MRI.^73^ We believe that fetal MRI has the potential to aid fetal medicine, and assist neonatal and paediatric surgical specialists in diagnosis, prognostication, parental counselling, and planning prenatal and postnatal care of babies with congenital anomalies.

## Supplementary Material

Supplementary Appendix

Supplementary video 1. Cervical lymphatic malformation (from figure 1A)

Supplementary video 10. Sacrococcygeal teratoma (from appendix p 3)

Supplementary video 11. Open spina bifida (from figure 5A)

Supplementary video 12. Open spina bifida (from figure 5A)

Supplementary video 13. Hydronephrosis (from appendix p 4, B)

Supplementary video 14. Multicystic dysplastic kidney (from appendix p 4, C)

Supplementary video 3. Congenital pulmonary airway malformation (from figure 2A)

Supplementary video 4. Congenital pulmonary airway malformation (from figure 2A)

Supplementary video 5. Bronchopulmonary pulmonary sequestration and (from figure 2B)

Supplementary video 6. Left congenital diaphragmatic hernia (from figure 2C)

Supplementary video 7. Left congenital diaphragmatic hernia (from figure 2C)

Supplementary video 8. Exomphalos and left congenital diaphragmatic hernia (from figure 4A)

Supplementary video 9. Exomphalos and left congenital diaphragmatic hernia (from figure 4A)

upplementary video 2. Oral teratoma (from figure 1B)

## Key messages

- MRI is commonly used in the classification of fetal brain anomalies; although its use in fetal body anomalies is less widely adopted, advances have led to the validation of its role in the antenatal investigation of several conditions
- Discrimination of soft tissues by fetal MRI enables delineation of anatomy in cases of fetal tumours, which aids antenatal counselling and might change management—eg, opting for an ex-utero intrapartum treatment procedure in fetal neck masses
- MRI volumetry can enable precise estimates of total lung volume, with a potential role in prognostication of congenital diaphragmatic hernia, congenital lung lesions, and anterior abdominal wall defects
- Fetal MRI is important in the investigation of fetuses with spina bifida: imaging of the fetal brain along with the spinal cord is an important factor in evaluating which patients could benefit from fetal surgery
- MRI has a limited role in the assessment of musculoskeletal and facial anomalies, which are best appreciated using two-dimensional or three-dimensional ultrasound; however, MRI assessment of the fetal airway shows its importance in complex anatomical assessment
- There are ongoing challenges in the antenatal diagnosis of several conditions, including anorectal malformation (visualisation of the fetal pelvis; absence of a dilated rectum in many cases) and oesophageal atresia (dilated upper pouch and visualisation of distal oesophagus) are challenging to diagnose on any form of antenatal imaging

## *Panel*: Imaging protocols and volumetric reconstruction

MRI provides excellent soft tissue contrast and is thus well suited for depicting the fetal visceral organs. The main disadvantage of MRI is that fetal motion and maternal breathing can disrupt image data during scanning. Fetal MRI acquisition protocols are therefore restricted to two-dimensional sequences, where stacks of single shot slices are rapidly acquired to freeze motion in time. The most common sequence used is T2-weighted half-Fourier acquisition single-shot turbo spin echo sequence because of its fast acquisition (less than 1 s per slice). Clinical protocols often employ thick slice acquisitions (3–5 mm) to increase signal-to-noise ratio and reduce acquisition time, at the expense of resolution. Even though sequences are standardised, image quality varies substantially owing to fetal size and position and the amounts of amniotic fluid and maternal adipose tissue.

The images presented in this Review were acquired with overlapping slices of reduced thickness (2·5 mm). The loss of continuity between adjacent slices because of motion (appendix p 1) can impede increasingly detailed characterisation of pathology; however, these stacks of densely spaced slices allow for high-resolution reconstruction of three-dimensional (3D) motion-corrected data (appendix p 1). The deformable slice-to-volume registration method allows for deformations of the fetal body, which enables not only the reslicing of the volume to any desired plane, but also segmentation, visualisation, and quantification of fetal organs in 3D as shown in this Review.

## Search strategy and selection criteria

We searched MEDLINE on Dec 2, 2020, for articles published in English, using the search terms “fetal MRI” and “antenatal MRI”. We also did extended searches of reference lists of included articles. If several articles illustrated a point, we selected the most important or recent of these articles, prioritising articles about the current capabilities and limitations of MRI in comparison to fetal ultrasound.
