# Supplementary material for: Fetal body MRI and its application to fetal and neonatal treatment: an illustrative review
Source: Lancet Child Adolesc Health. Author manuscript; Available in PMC 2023 Feb 8. (PMC7614154; doi:10.1016/S2352-4642(20)30313-8)
Supplement: Supplementary Appendix [file EMS157888-supplement-Supplementary_Appendix.pdf]

THE LANCET
Child & Adolescent Health

Supplementary appendix

This appendix formed part of the original submission and has been peer reviewed. We post it as supplied by the authors.

Supplement to: Davidson JR, Uus A, Matthew J, et al. Fetal body MRI and its application to fetal and neonatal treatment: an illustrative review. *Lancet Child Adolesc Health* 2021; published online March 12. http://dx.doi.org/10.1016/S2352-4642(20)30313-8.

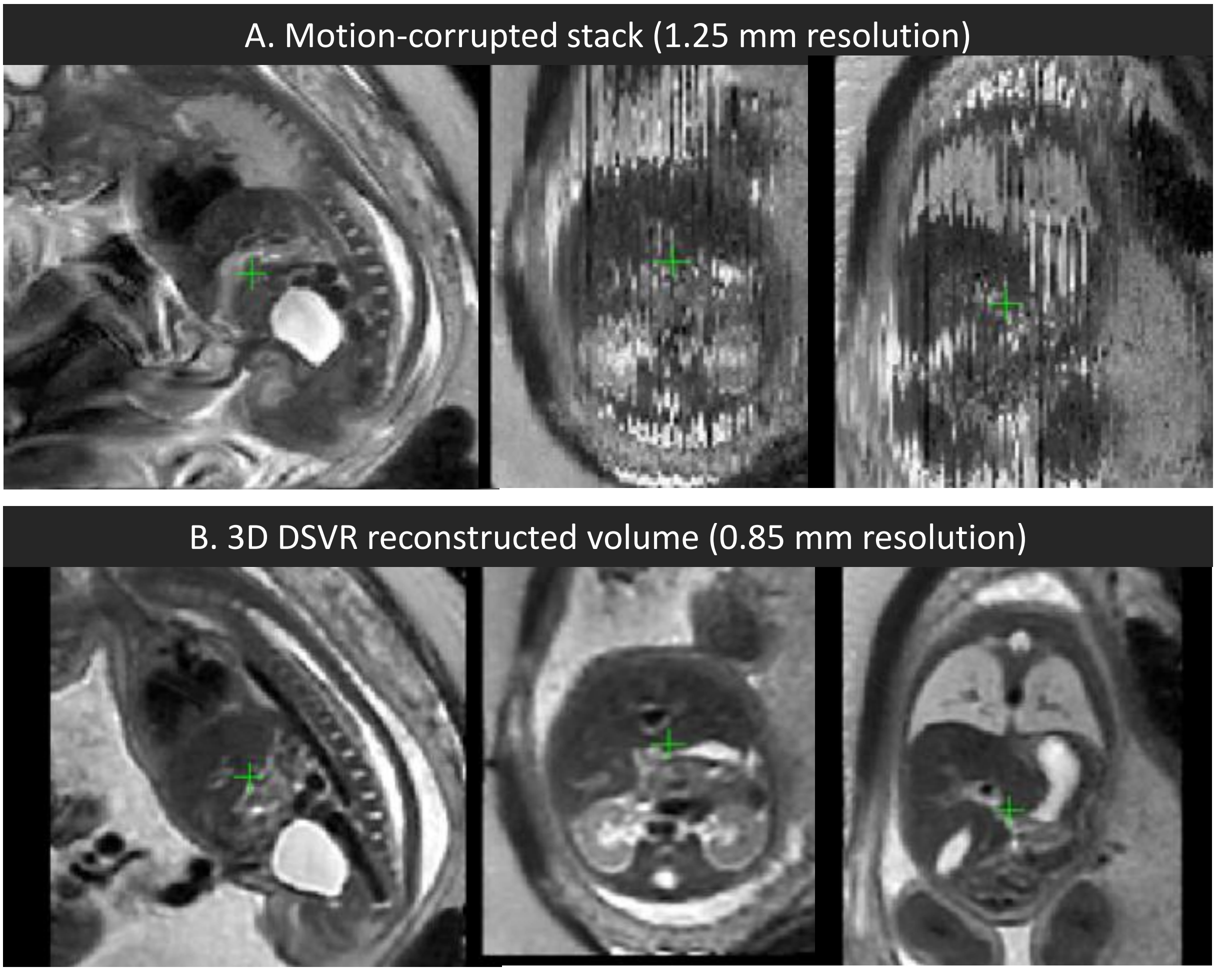

Supplemental Figure S1.
A: An example of in-utero MRI scan of a normal control fetus at 29+3 weeks GA: original acquisition plane and through-plane orthogonal views of an acquired stacks of slices. Uncontrollable fetal motion during acquisition lead to loss of anatomical continuity between adjacent 2D slices and corruption of volumetric information in 3D stacks.
All MRI datasets of the cases investigated in the review were acquired on Phillips Achieve 1.5T scanner, HASTE sequence, resolution 1.25x1.25x2.5 mm, slice gap -1.25 mm, TE=80-100 ms, TR = 15 s, acquisition time approximately 80s per stack. Each dataset contained 6-9 stacks of various acquisition planes. B: Motion-corrected 3D volume (0.85 mm isotropic resolution) reconstructed using DSVR method[5] from multiple stacks.

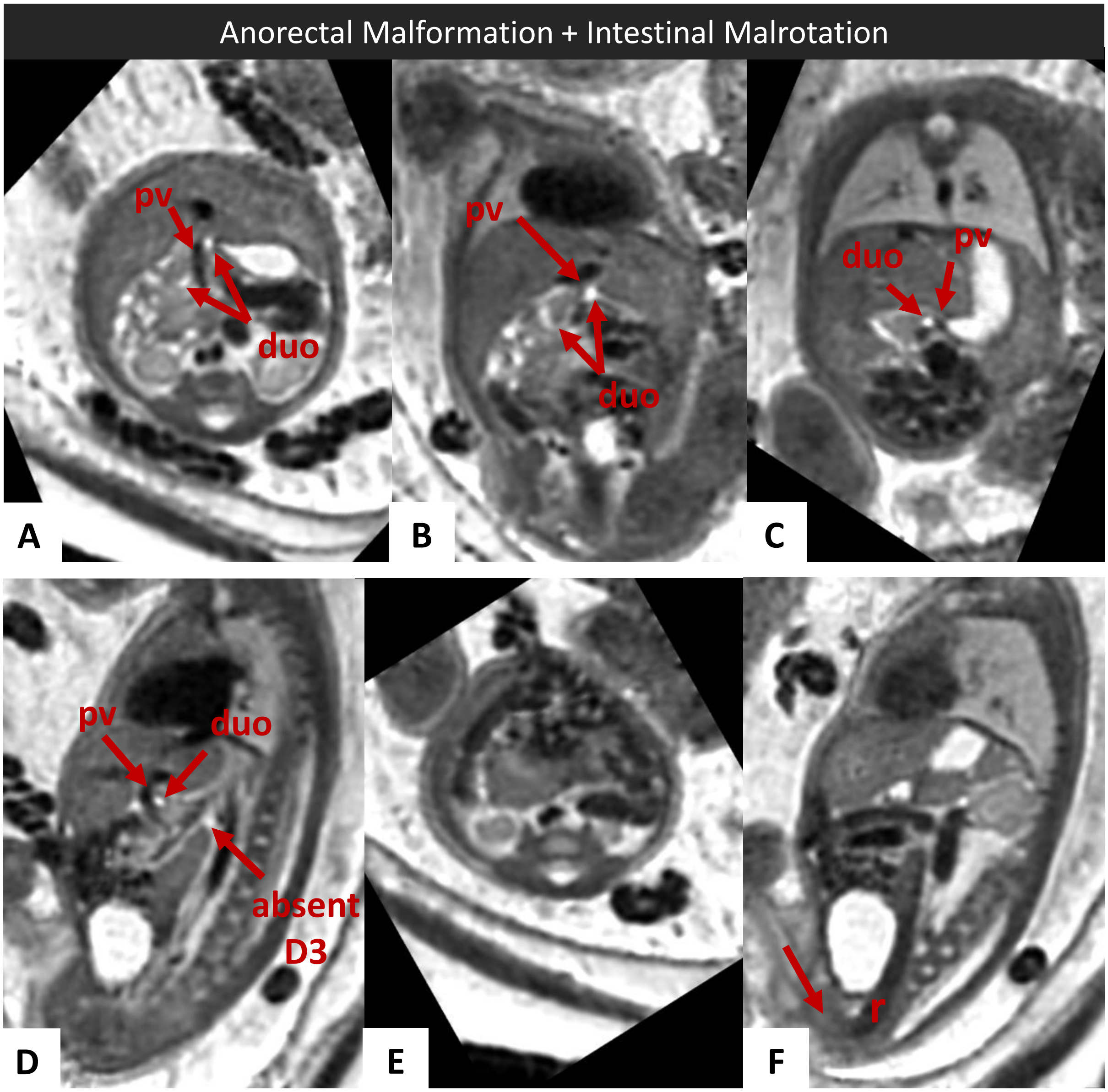

Supplemental Figure S2.
Representative DSVR-reconstructed MRI of a 31+1 week fetus. A-C: Portal vein (pv) can be seen passing anteriorly to the duodenum (duo) which lacks a retroperitoneal course (absent D3, image D). E: Meconium signal in the fetal colon is only seen on the left of the abdomen. F: The rectum (r) is seen to possibly deflect anteriorly (arrow).

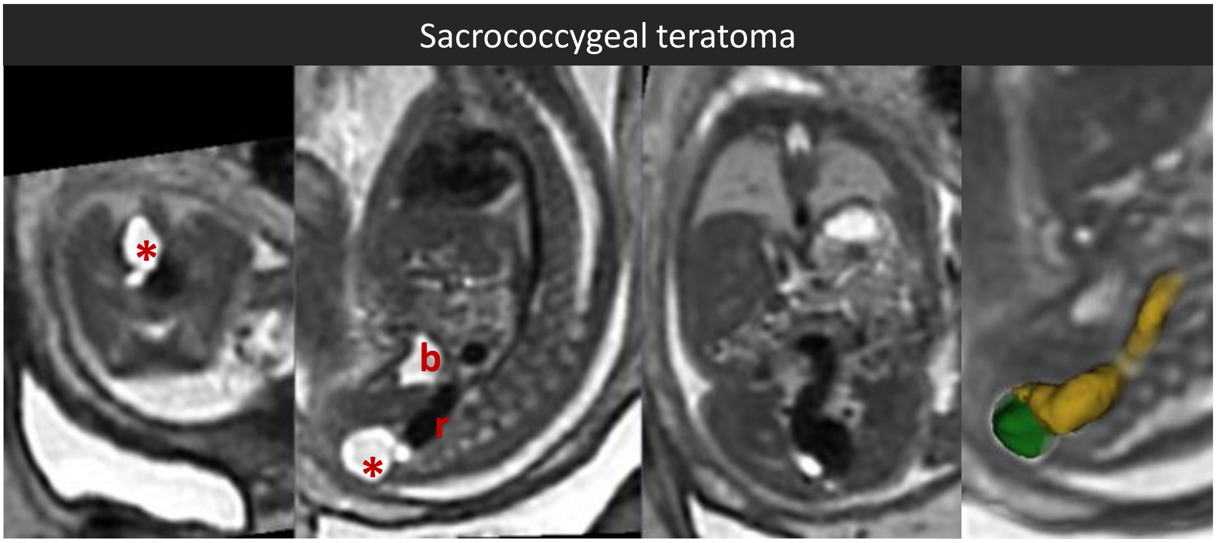

Supplemental Figure S3.
Complex cystic structure (asterisk *) is appreciated in the pelvic area, consistent with the cystic component of an intrapelvic sacrococcygeal teratoma (type IV lesion). The lesion is adjacent to the low-signal, meconium-filled rectum (r) and the bladder (b) is seen more anteriorly and superiorly within the pelvis. 3D reconstruction demonstrates the rectum in yellow and the cystic component of the sacrococcygeal teratoma in green.

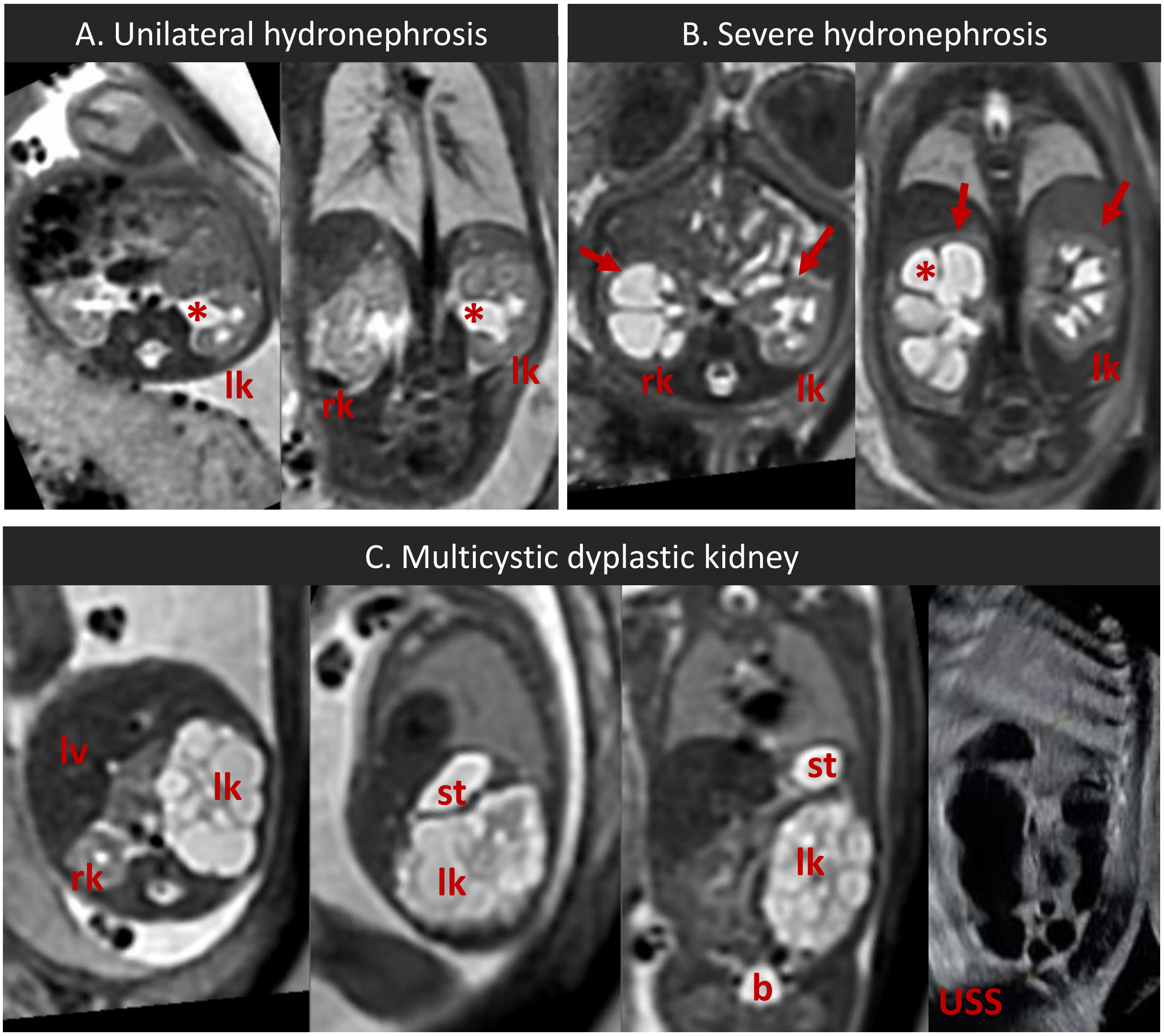

Supplemental Figure S4.
Lesions of the kidney and urinary tract in three separate patients, all showing left (lk) and right (rk) kidneys. A+B both demonstrate calyceal dilatation(*) and parenchymal thinning (arrows). C: Liver (lv), stomach (st) and bladder (b) are also shown along with the multicystic left kidney.
